# A Likelihood-Based Framework for Variant Calling and *De Novo* Mutation Detection in Families

**DOI:** 10.1371/journal.pgen.1002944

**Published:** 2012-10-04

**Authors:** Bingshan Li, Wei Chen, Xiaowei Zhan, Fabio Busonero, Serena Sanna, Carlo Sidore, Francesco Cucca, Hyun M. Kang, Gonçalo R. Abecasis

**Affiliations:** 1 Center for Human Genetics Research, Department of Physiology and Biophysics, Vanderbilt University, Nashville, Tennessee, United States of America; 2 Department of Pediatrics, Children's Hospital of Pittsburgh, Pittsburgh, Pennsylvania, United States of America; 3 Center for Statistical Genetics, Department of Biostatistics, School of Public Health, University of Michigan, Ann Arbor, Michigan, United States of America; 4 Istituto di Ricerca Genetica e Biomedica, Consiglio Nazionale delle Ricerche (CNR), Monserrato, Italy; Georgia Institute of Technology, United States of America

## Abstract

Family samples, which can be enriched for rare causal variants by focusing on families with multiple extreme individuals and which facilitate detection of *de novo* mutation events, provide an attractive resource for next-generation sequencing studies. Here, we describe, implement, and evaluate a likelihood-based framework for analysis of next generation sequence data in family samples. Our framework is able to identify variant sites accurately and to assign individual genotypes, and can handle *de novo* mutation events, increasing the sensitivity and specificity of variant calling and *de novo* mutation detection. Through simulations we show explicit modeling of family relationships is especially useful for analyses of low-frequency variants and that genotype accuracy increases with the number of individuals sequenced per family. Compared with the standard approach of ignoring relatedness, our methods identify and accurately genotype more variants, and have high specificity for detecting *de novo* mutation events. The improvement in accuracy using our methods over the standard approach is particularly pronounced for low-frequency variants. Furthermore the family-aware calling framework dramatically reduces Mendelian inconsistencies and is beneficial for family-based analysis. We hope our framework and software will facilitate continuing efforts to identify genetic factors underlying human diseases.

## Author Summary

New sequencing methods can be used to study how genetic variation contributes to disease. For studies of rare variation, family designs are especially attractive because they allow even very rare variants to be observed in multiple individuals and because they can be used to study the impact of *de novo* mutation events. An important challenge is that most raw sequencing data include many errors. Here, we develop a new approach for interpreting sequence data. We show that by analyzing sequence data across many family members together it is possible to greatly reduce error rates (measured either as the number of true variants that are missed or the number of false variants that are claimed). In addition to facilitating detection and genotyping of SNPs, our methods can interface with existing tools to improve the accuracy of more challenging short insertion deletion polymorphisms and other types of variants. Our methods should make studies of families even more attractive because, in addition to making it easy to study rare variants and *de novo* mutation events, family studies will now be able to better transform sequence data into accurate genotypes.

## Introduction

Next generation sequencing (NGS) technologies are being used to identify and genotype rare, low frequency and common genetic variants in a variety of settings. The 1000 Genomes Project is generating a publicly available and increasingly comprehensive catalog of human variation [1]. Whole exome sequencing studies are accelerating the rate of discovery for rare Mendelian disease associated variants [2]–[5]. Rare variants are also expected to play an important role in complex disorders [6]–[8] and several large scale NGS studies of complex diseases are underway.

Family designs provide a promising approach for these NGS studies [7]–[9]. Samples of families with multiple affected individuals can be enriched for causal variants, increasing the power of rare variant association studies compared to studies of unrelated cases and controls [10], [11]. Furthermore, families can also be useful in studies that initially examine unrelated individuals. When a rare variant of interest is identified, sequencing family members can help identify additional carriers, facilitating validation of findings. Finally, by modeling inheritance of alleles within families greater accuracy of variant calling may be achieved, mitigating the effects of sequencing error associated with many NGS platforms. Also important, families allow explicit identification of *de novo* mutations, which are important contributors to some of complex diseases [12]–[20]. Although some *de novo* events, including copy number variants in particular have been extensively studied using microarrays [12], [14], [15], next generation sequencing will facilitate examination of other types of *de novo* events, including point mutations [13], [16]–[20].

Here, we focus on a critical step in the analysis of next generation sequence data, which is the identification of variant sites in a sample and estimation of genotypes for each individual being studied [21], [22]. This process is challenging due to sequencing errors, potential allelic drop-out when coverage is low, and errors in read mapping, among others. Although much work has been done on the identification of variant sites and genotype calling using next generation sequence data, most prior work has focused on the analysis of unrelated individuals and did not allow for explicit modeling of relationships between sequenced individuals. We develop and implement a likelihood based framework that considers sequence data for all individuals in a family jointly. Our framework (implemented in our software for Polymorphism and Mutation discovery - PolyMutt) builds on the Elston-Stewart peeling algorithm [23]–[25], and provides a probabilistic framework for evaluating the evidence for specific genetic variants, individual genotypes and *de novo* mutation events. Through simulations, we show that by modeling family relationships our framework improves variant detection and genotype calling, including detection of *de novo* mutation events. Specific advantages of our model include a modest increase in the number of variant sites detected, a marked improvement in genotype accuracy, greatly improved specificity for lists of potential *de novo* mutation events, and elimination of most Mendelian inconsistencies due to genotyping error. Overall, our work provides analysis tools and study design guidance for investigators wishing to include families in their NGS sequencing studies.

## Methods

### Genotype likelihood calculation

For simplicity, we first describe how our methods can be applied to SNPs. In a later section, we describe the modifications required to apply our methods to call short insertion and deletion (indel) polymorphisms. Suppose for a sequenced individual a genome position is covered by *N* mapped *A*, *C*, *G* and *T* bases with counts of *N_A_, N_C_, N_G_* and *N_T_* respectively. For each of these mapped bases 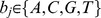 an error rate 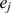 is provided to indicate the probability that the base is incorrectly called. Let *R* denote the base calls overlapping the position of interest across all aligned read data in an individual. Due to sequencing errors and allele dropout all 10 genotypes are possible, and genotype likelihoods [26] (GL) are calculated to quantify the likelihood of *R* for each underlying true genotype. Genotype likelihoods are defined as 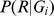, *i* = 1,…,10, where *G_i_* is one of *AA, AC, AG, AT, CC, CG, CT, GG, GT or TT*, given the error rate for each base. As an example, the GL for genotype *AA* is 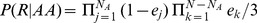 where 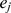 is the estimated error rate for the *j* ^th^ A base and 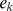 is the error rate of the *k* ^th^ non-*A* base. This assumes each base has an equal probability of being miscalled as any of the three alternative bases. For heterozygous *AC* genotypes, we assume each base originates from either the *A* or *C* allele with equal probability, that is 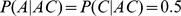. Then for the *j* ^th^ base 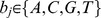 the GL is
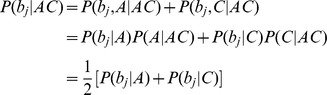
If the base *b_j_* is either *A* or *C* then 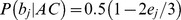 and otherwise 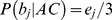. Then the GL of genotype *AC* combining all bases is 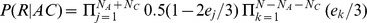 where 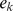 is the estimated error rate of the *k* ^th^ sequence base which is neither *A* nor *C*. The calculation assumes all base calling errors are independent – see the work of Li and Durbin [26] for an alternative. The GL calculation is similarly carried out for each potential genotype. Usually the 10 GL values are stored in binary Genotype Likelihood Format (GLF) [22], [26] files or in Variant Call Format (VCF) [27] files. In some cases, these likelihoods may be improved by explicitly modeling reference biases due to alignment artifacts.

### Variant calling in families—likelihood of reads in pedigrees

Let **R** *^j^* and **G** *^j^* denote respectively the vector of aligned bases at a site and the vector of genotypes for all individuals in the *j* ^th^ family. Given the error rate of each base the likelihood of sequence data for this family is
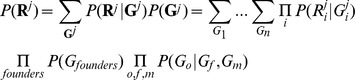
In the above calculation it is assumed that in a family the aligned bases for each individual depend only on this person's genotypes so that 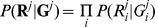. The likelihood calculation involves a nested summation of joint likelihood of reads and genotypes over all possible genotype configurations for the pedigree. For each genotype configuration the joint likelihood consists of product of three parts: (1) the likelihood of aligned bases conditional on each individual genotype, 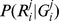, (2) the probability of founder genotypes 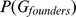 and (3) the probability of offspring genotypes conditional on their parental genotypes, 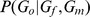, for all parent-offspring triplets. For multiple families the likelihood is 
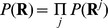
 which is the product of the likelihoods for individual families. In calculating the likelihood of reads in a pedigree, the genotype likelihood 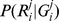 is typically retrieved from the GLF or VCF file for the *i* ^th^ individual in the *j* ^th^ family, generated as described above or using one of several existing approaches [21], [26]. Founder genotype frequencies 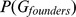 are usually unknown and can be estimated by maximum likelihood from the data [28]. In this study we assumed that variant sites are bi-allelic and in Hardy-Weinberg equilibrium. We used the Brent optimization algorithm [29] to estimate the allele frequency by maximizing 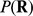 with respect to allele frequencies and to obtain the maximum likelihood 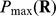. Note that this approach uses all available information to estimate founder allele frequencies (for example, when founders are not sequenced, their frequencies can be estimated based on observed alleles in their offspring).

### Variant calling in families—variant site discovery

A key step in the analysis is to determine which sites vary, and (for these variant sites) to identify the segregating alleles. For bi-allelic variants we find the two alleles, *A_1_,A_2_*, with the maximal posterior probability 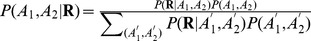. For a given two-allele configuration the probability of observing the reads 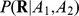 can be obtained as 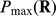 above. The configurations we considered include monomorphism where all alleles match the reference (*A_1_* = *A_2_* = *A_ref_*), the transition from the reference, the two possible transversions from the reference, and other less likely but possible configurations where neither of the alleles in the sequenced families is the same as the reference (because the reference allele is incorrectly called or very rare). From classic coalescent theory [30], in a sample with *N* diploid founders and in the absence of natural selection, the prior probability that a site includes non-reference alleles is 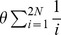 where 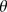 is the population scaled mutation rate per site and is set to 1/1000 in this study. If a site varies, we set the probability that the mutation is a transition from the reference at ∼2/3 and the probability of a transversion from the reference is ∼1/6 (there are two possible transversions at each site). These prior probabilities are biologically meaningful and consistent with the estimates in dbSNP and the 1000 Genomes Project data [1]. To summarize confidence in each variant site we define a Phred-like variant quality score 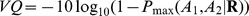.

### Variant calling in families—genotype calling

For a variant site, the steps described in the previous section provide the two most likely alleles and the corresponding founder allele frequency. Genotypes for each individual can be inferred conditioning on available sequence data for all individuals in a family. We calculate the posterior probabilities of genotypes for individual *i* in family *j*, given the read data in a family as follows:
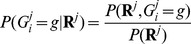
The numerator 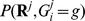 can be calculated analogously to 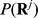 by considering only terms where 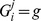. To call the genotype of an individual, we record the modal genotype (i.e. the one with the highest posterior probability) and the expected alternative allele count (dosage). The expected allele count is a real number between 0 and 2 which summarizes the expected number of non-reference alleles in an individual and which can be used in downstream phenotype association analyses [31]. We define a genotype quality score for each genotype as 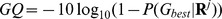. This framework considers sequence data at each variant site independently, and does not account for LD information (which could further improve calling accuracy) – for an example of how linkage disequilibrium information can be used in trios, see [32].

Note that the likelihood calculation, variant site discovery procedure and genotype calling strategy above apply to unrelated individuals as well as families, or to mixed samples of unrelated and related individuals. When comparing the performance of variant calling using family information with standard approaches that ignore relatedness, we use the likelihood framework above but analyze all sequenced individuals twice, first, modeling the correct relationships, and second, treating all individuals as if they were “unrelated”.

### Integration of *de novo* mutation detection

In our likelihood calculations so far, we have assumed Mendelian transmission. To integrate the detection of *de novo* mutations in the same framework, the transmission probabilities from parents to offspring are modified to allow for *de novo* mutation events. Assuming mutation rate 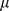 for the site of interest, then the mutation model is defined as a transition matrix from parental alleles to mutant alleles in an offspring, for example:
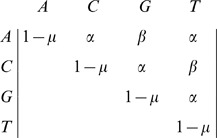
This simple model assumes that a parental allele can be mutated into any of the other 3 alleles, transitions and transversions have mutation rates of α and β respectively and *μ* = 2α+β. Our implementation allows for alternative, and arbitrary, user supplied mutation matrices. In this model, a non-founder can have any of the 10 possible genotypes irrespective of parental genotypes (naturally, most combinations that include alleles not present in the parents will be *a priori* unlikely when the mutation rate estimate is low). Using the updated definition of transmission probabilities, the likelihood function of reads in a family allowing for *de novo* mutation events can be calculated and is denoted as *L_denovo_* Except for the updated transmission function, our likelihood, site discovery and genotyping proceed exactly as before. To assess the evidence of a *de novo* mutation, the likelihood disallowing *de novo* mutation, denoted as *L_mendel_*, is also calculated and the likelihood ratio 
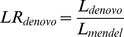
, or equivalently the posterior probability of *de novo* mutation 
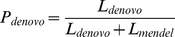
, indicates the confidence in *de novo* mutation detection.

### Genotyping refinement on insertion and deletion variants

Calculation of genotype likelihoods for insertion and deletion polymorphisms (indels) calling usually involves re-alignment of each read to candidate indel haplotypes [33] and is an area of very active research. By using standard formats, like the Variant Call Format (VCF), our implementation can use genotype likelihoods for any type of polymorphism – as calculated by the Genome Analysis Toolkit (GATK) [34], [35] or samtools [26], [36], for example. This means that any improved methods of genotype likelihood calculation for indels or other classes of polymorphism can be combined with our modeling of familial segregation. To illustrate the possibilities, we used genotype likelihoods calculated by the GATK as a starting point for calling of indel genotypes, taking family inheritance into account.

### Simulations

To simulate sequence data, we first simulated 100 independent 1 Mb regions each with 10,000 haplotypes based on previously proposed genetic models for European and African ancestry samples [37]. For each pedigree, founder genotypes were generated by randomly pairing haplotypes from this sample. For descendant individuals, crossovers were simulated according to a constant recombination rate of 1.5×10^−8^ and recombinants were randomly transmitted to offspring. For an individual genome with coverage *c*, we assumed that the number of bases covering a position follows a Poisson distribution with mean *c*. To simulate bases, we first randomly drew a number from *Poisson(c)* at a site for an individual, assuming that each base originated with equal probability from either allele. Then each base 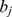 was simulated with error rate 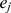: with probability 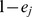 the base matched the underlying template allele and with probability 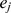 the observed base was a sequencing error, and one of the three alternative bases was sampled with equal probability. The GL was calculated as described in the section “*Genotype likelihood calculation”* after all bases were generated. This process was repeated for all sites and genotype likelihood values for each individual were stored in GLF files [22].

To assess the power of this framework for *de novo* mutation detection, we started with the simulation framework above, and then introduced *de novo* mutations at a rate of 1.4×10^−8^ per site per generation [38]. To evaluate false positive *de novo* mutation calls, the same calling algorithm was applied to sequencing data simulated without *de novo* mutations.

Note that the above simulation framework assumes that the mapping is 100% correct. To evaluate the performance in the presence of mapping errors for both SNPs and short indels, we used simulations that mimic the read mapping protocols used in current next-generation sequencing studies. First, we randomly selected CEU samples from the 1000 Genomes Project [1] as founders and generated founder haplotypes from the VCF file for the March 16, 2012 Phase I haplotype release. Non-founder haplotypes were then generated simulating recombination and segregation of founder haplotypes through the pedigree, as above. Then, 100-bp long paired-end reads with an average insert size of 400 bp and standard deviation of 50 bp were randomly simulated for each person, assuming a Poisson distribution for read start points, a normal distribution for insert sizes, and a per base Phred-scaled quality of Q20 (corresponding to a 1% error rate). Simulated paired-end reads were aligned to the reference genome using BWA [39] and then GATK [34], [35] was used to perform indel re-alignment and base quality re-calibration. The list of known indels was provided to GATK for re-alignment prior to SNP and indel calling.

**Figure 1:**
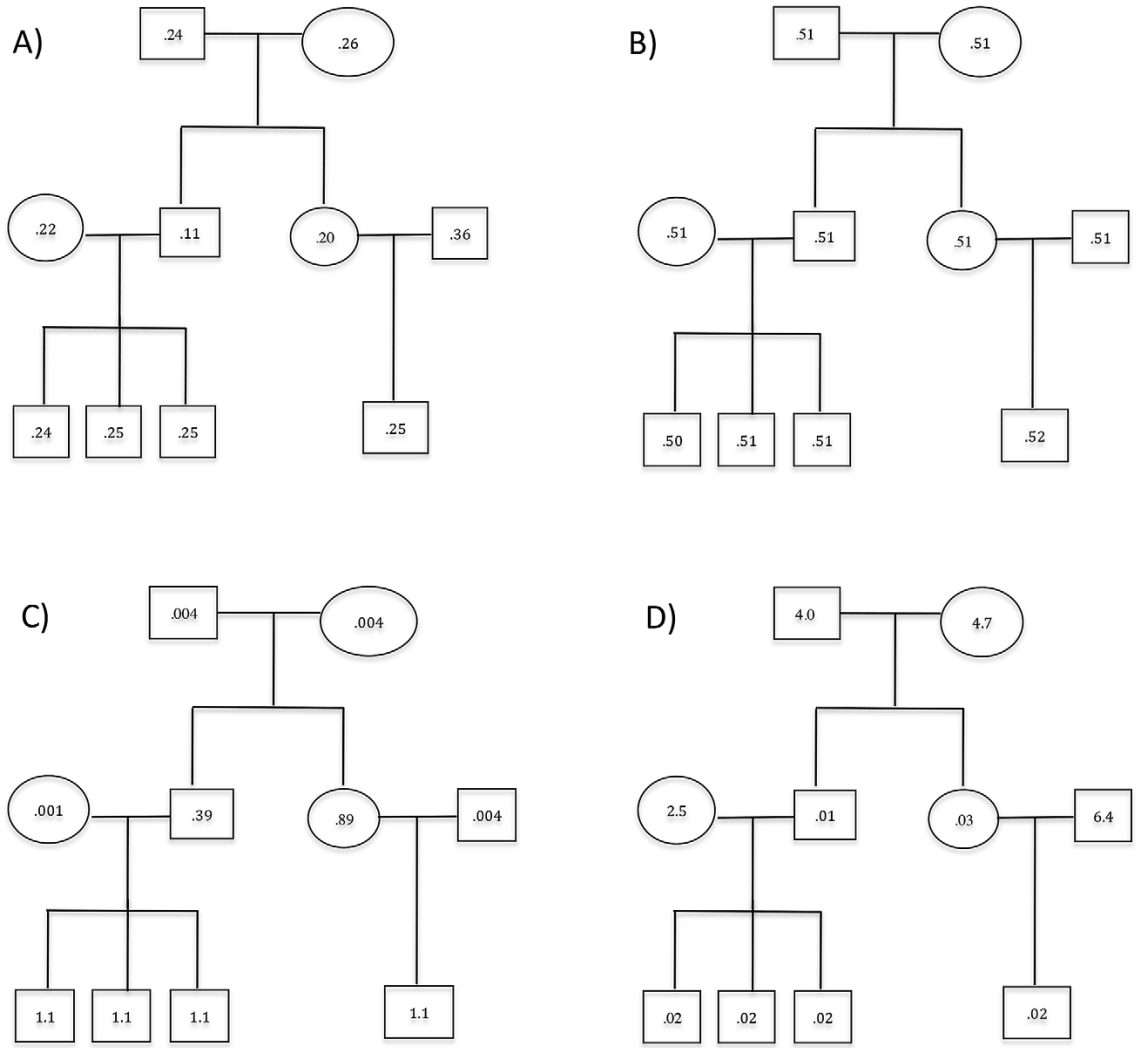
Three-generation extended pedigrees. A) is a 3-generation extended pedigree with numbers labeling the individual heterozygous genotype mismatch rates (%) at coverage of 15× with base quality of Q20 without mapping error and panel B) labels the corresponding mismatch rates for the standard approach of ignoring relatedness. Panel C) and D) display the heterozygous mismatch rates (%) when a fixed sequencing effort of 150× is allocated differently to family members: Panel C) is for the situation where the founders are allocated 30× while non-founders have 5× and in Panel D) founders and non-founders have coverage of 6× and 21× respectively.

The family structures we simulated include trios, sibships, nuclear families and 3-generation extended pedigrees. Since we used only one extended pedigree (shown in Figure 1), we will use “3-generation pedigree” throughout to denote this extended pedigree. Variant calling and *de novo* mutation detection were evaluated for sequencing coverage of 5×–40× and base quality scores of Q20 and Q30 which correspond to per base sequencing error rates of 0.01 and 0.001, respectively. Due to the computational demands of read mapping, we carried out simulations based on the 1000 Genomes data only for the 3-generation pedigree and for trios.

## Results

### Non-reference genotypes identified per individual

We calculated the percentage of non-reference genotypes not discovered (i.e. false negative rate) per individual in various pedigrees for joint family calls and for a standard analysis that ignores family structure. For all sequencing quality scores and coverage, the 3-generation pedigree has the lowest rate of missed non-reference genotypes (Table 1). For nuclear families, the lowest rate of missed non-reference genotypes was observed in families where both parents and 2 siblings were sequenced (Table 1). Other configurations with 4 sequenced individuals per nuclear family, such as those where one parent and three offspring were sequenced or where four siblings were sequenced had higher rates of missed non-reference alleles (among these two configurations, the proportion of missing genotypes was highest when no parental genotypes were available) (Table 1). This demonstrates that sequencing parents can be beneficial, reducing the rate of missed variant sites. Although increasing sequencing quality increases the fraction of variant genotypes discovered, increasing coverage has a far more dramatic effect. For example, the false negative rate is 3.16% for sequencing at 5× coverage in the 3-generation pedigrees when bases are simulated with Q20. This rate is reduced to 2.67% when base quality increases to Q30 and reduced to 0.51% when coverage is doubled (i.e. 10×) (Table 1). In all cases, when the variant calling was performed using the standard approach that assumes all sequenced individuals are unrelated, higher proportions of variant genotypes were missed (Table 1). The false negative rates reported for the standard approach were averaged across all individuals in all pedigrees since the missing rates are similar for different pedigrees.

**Table 1:** Percentage of missing non-reference genotypes (i.e. false negatives) per individual in families for variants called by joint modeling family data and the standard approach of ignoring relatedness for sequencing coverage between 5× and 30× and for input sequence data with Phred-scaled quality of 20 (error rate of 1% per base) or 30 (error rate of 0.1% per base) without mapping error.

		False negative rate per individual (%)				
Base Quality	Family structure	5×	10×	15×	20×	30×
20	3-generation families	3.16	0.51	0.114	0.0260	0.00152
	2 parents+2 siblings	3.53	0.57	0.123	0.0267	0.00156
	1 parent+3 siblings	4.15	0.68	0.152	0.0341	0.00194
	0 parents+4 siblings	4.23	0.73	0.160	0.0362	0.00204
	Standard approach	5.52	1.08	0.234	0.0531	0.00290
30	3-generation families	2.67	0.27	0.046	0.0087	0.00027
	2 parents+2 siblings	2.96	0.30	0.049	0.0094	0.00028
	1 parent+3 siblings	3.70	0.42	0.064	0.0128	0.00036
	0 parents+4 siblings	3.85	0.47	0.069	0.0134	0.00045
	Standard approach	4.72	0.57	0.096	0.0180	0.00055

For all scenarios 300 sequenced individuals were simulated.

### Genotype accuracy

We compared the best-guess genotype calls for each individual to the true simulated genotypes and estimated the mismatch rates for all genotypes (All) and for 3 sub-categories classified based on true genotypes: homozygous reference allele (HomRef), heterozygotes (Het) and homozygous alternative allele (HomAlt). For different pedigrees and genotype categories, the trend for mismatch rates again showed that examining the 3-generation pedigrees provided the most accurate results while examining siblings with no parents provided the least accurate option (Table 2). Among all genotype categories, heterozygotes are the most difficult to call correctly and the mismatch rates for this category can be substantially larger than for the others. For example at 15× with base quality Q30, heterozygotes in siblings have a mismatch rate >3 times larger than HomAlt genotypes and >20 times larger than HomRef genotypes (Table 2). Similar to the power of detecting variant genotypes, coverage has a more dramatic effect than sequencing quality on genotype accuracy and at 30× all categories of genotypes can be reliably identified (Table 2). When relatedness was ignored, the genotypes were less accurate for all categories and for some pedigrees the mismatch rates could be substantially increased. For example, the mismatch rate for heterozygotes called using the standard approach that ignores family structure was typically about 2 times higher than for heterozygotes called using the full 3-generation pedigrees (Table 2).

**Table 2:** Genotype mismatch rates (%) for different family structures with sequencing coverage of 5×, 15×, and 30× and input bases with Phred-scaled quality Q20 (1% error rate) or Q30 (0.1% error rate) without mapping error.

		Genotype mismatch rate (%)											
		5×				15×				30×			
Base Quality	Family structure	All	HomRef	Het	HomAlt	All	HomRef	Het	HomAlt	All	HomRef	Het	HomAlt
Q20	3-generation families	1.69	0.42	7.09	2.25	0.0546	0.0156	0.2423	0.0756	6.8*10^−4^	2.6*10^−4^	2.5*10^−3^	1.3*10^−3^
	2 parents+2 siblings	1.80	0.45	7.77	2.53	0.0562	0.0160	0.2617	0.0872	7.0*10^−4^	2.7*10^−4^	2.7*10^−3^	1.5*10^−3^
	1 parent+3 siblings	2.12	0.49	9.19	3.02	0.0723	0.0220	0.3265	0.1151	9.9*10^−4^	4.0*10^−4^	3.7*10^−3^	1.8*10^−3^
	0 parents+4 siblings	2.33	0.59	9.45	4.02	0.0814	0.0251	0.3526	0.1539	1.1*10^−3^	4.6*10^−4^	4.0*10^−3^	2.2*10^−3^
	Standard approach	2.88	0.62	12.39	4.10	0.1098	0.0292	0.5082	0.1806	1.5*10^−3^	5.2*10^−4^	6.0*10^−3^	2.6*10^−3^
Q30	3-generation families	1.27	0.22	6.33	1.49	0.0215	0.0057	0.0997	0.0289	1.1*10^−4^	2.7*10^−5^	5.9*10^−4^	9.4*10^−5^
	2 parents+2 siblings	1.35	0.24	7.02	1.70	0.0217	0.0057	0.1057	0.0313	1.2*10^−4^	2.6*10^−5^	6.8*10^−4^	1.2*10^−4^
	1 parent+3 siblings	1.67	0.24	8.83	1.98	0.0288	0.0077	0.1385	0.0393	1.6*10^−4^	3.6*10^−5^	7.7*10^−4^	2.8*10^−4^
	0 parents+4 siblings	1.81	0.28	9.16	2.53	0.0335	0.0084	0.1659	0.0485	1.9*10^−4^	3.8*10^−5^	9.9*10^−4^	3.0*10^−4^
	Standard approach	2.14	0.21	11.64	2.46	0.0448	0.0105	0.2242	0.0592	2.5*10^−4^	5.7*10^−5^	1.3*10^−3^	3.4*10^−4^

The mismatch rates are shown for 4 genotype categories: all genotypes (All), homozygous reference allele (HomRef), heterozygotes (Het), and homozygous alternative allele (HomAlt).

### Genotype accuracy by frequency

**Figure 2:**
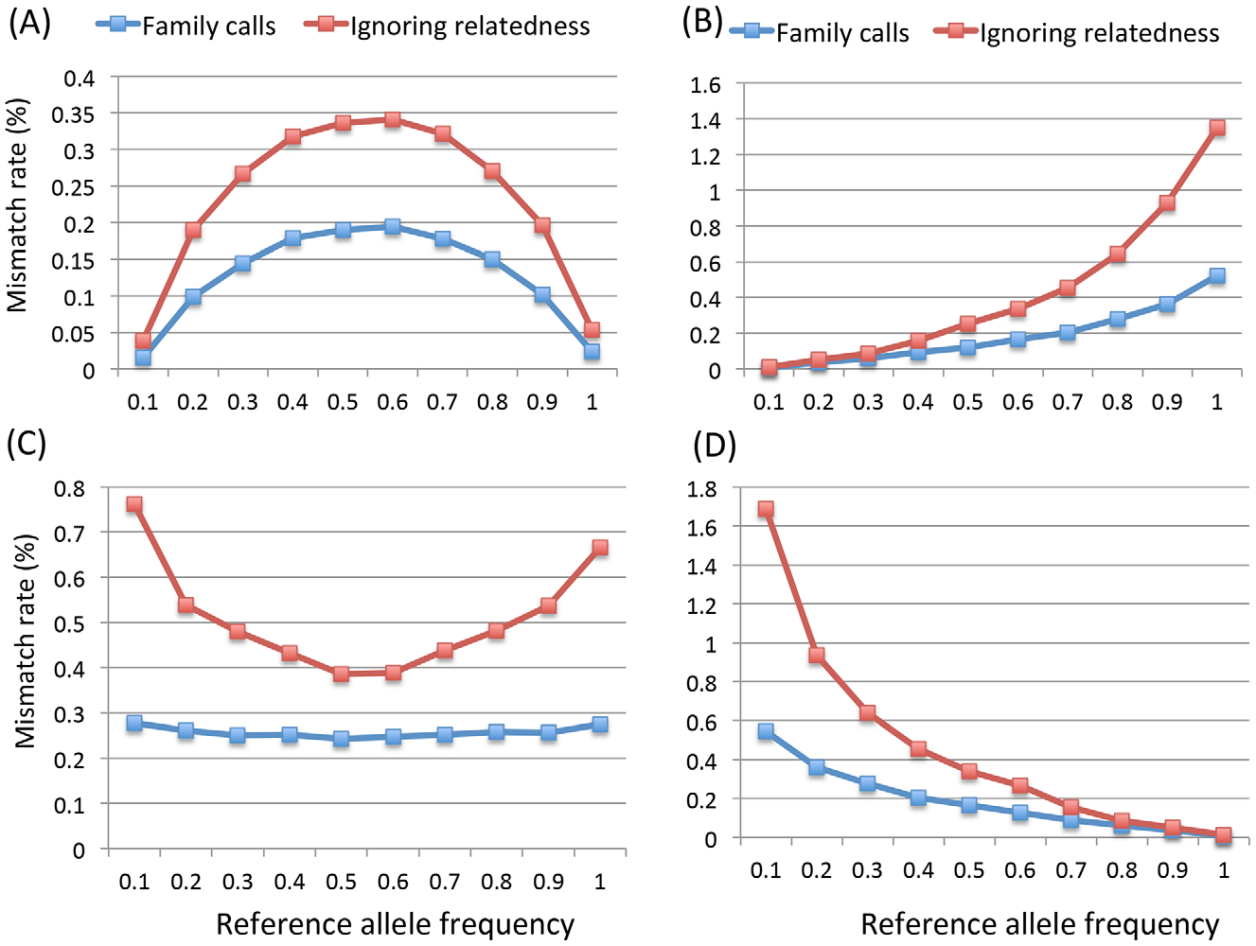
Mismatch rates (%) of 4 categories of genotypes by the reference allele frequencies for pedigrees of quartet (two siblings and their parents) with base quality Q20 at 15× without mapping error. The 4 categories are (A) overall genotypes, (B) homozygous alternative allele, (C) heterozygotes and (D) homozygous reference allele.

We further investigated the genotype accuracy as a function of the reference allele frequency in pedigrees with both parents and 2 siblings sequenced with ∼15× coverage and Q20 bases. Considering all genotypes, the mismatch rate is lower at low minor allele frequencies (MAF) and increases when MAF becomes higher (Figure 2A). Different sub-categories of genotype show markedly different patterns of mismatch rates as a function of allele frequency (Figure 2B, 2C, 2D); in general, error rates are higher for rare genotypes than for common ones. For example, HomRef genotypes have lower mismatch rate for high reference allele frequencies and higher mismatch rates for low reference allele frequency values (Figure 2D). The pattern is the opposite of that for HomAlt genotypes (Figure 2B). Figure 2B and 2D are not exact mirror images because of systematic differences between reference and non-reference alleles in both our simulation (where reference alleles are typically more common) and our analysis strategy (where very rare alternate sites might not be discovered at all, and thus not contribute to genotype accuracy calculations). For heterozygotes the pattern is roughly symmetric relative to allele frequency and heterozygotes are easiest to call when allele frequencies are close to 0.5 (Figure 2C). When the standard approach was applied, genotype mismatch rates for all categories dramatically increased, especially at lower MAF (Figure 2). For example at MAF below 0.1, the mismatch rates of HomRef and HomAlt genotypes in the standard approach are about 2–3 times higher than when family relationships are modeled (Figure 2B, 2D). These observations suggest that it is critical to jointly model family relationships explicitly, particularly when calling genotypes for rare variants which are usually of great interest for sequence based association studies.

### Individual genotype accuracy in three-generation extended pedigrees

The genotype mismatch rates discussed above are averaged over all individuals in a family and we next examined the genotype accuracy for different members of the simulated 3-generation extended pedigrees shown in Figure 1, focusing results on heterozygous genotypes. Individual II-2 has the lowest mismatch rates (Figure 1A), mainly due to the strong familial inheritance constraints imposed on this individual by multiple sequenced offspring and sequenced parents. On the other hand, individual II-4, with a single sequenced offspring, has the highest mismatch rate due to the weakest Mendelian inheritance constraints in the pedigree (Figure 1A). Other individuals fall in between and individual error rates are roughly consistent with the degree of inheritance constraints (Figure 1A). When the relatedness was ignored, the individual genotype accuracy was jeopardized with similar but much elevated mismatch rates for all family members (Figure 1B). The greatest loss is observed for individual II-2 whose mismatch rate increased from 0.11% to 0.51% (Figure 1).

### Mendelian inconsistency

Ignoring relatedness typically increased the rate of Mendelian inconsistencies, defined as the number of incompatible genotypes per triplet (father, mother and offspring), by >>100-fold (Table 3). For example, at 10× with base quality Q20, the Mendelian inconsistency rate is 1.9*10^−6^ when family structure is modeled explicitly and 4.9*10^−3^ when treating individuals as unrelated (Table 3). Even at higher coverage and base quality scores, the rate of Mendelian inconsistencies in genotypes called without knowledge of family structure remains high (Table 3). Excluding markers with Mendelian inconsistences has the risk of discarding true signals. Importantly, in studies that aim to detect *de novo* mutations, erroneous genotype calls suggesting Mendelian inconsistencies can become false candidate sites for *de novo* mutation events and lead to wasted replication resources. Therefore it is again advantageous to jointly model family data to infer genotypes – reducing the number of false positive *de novo* variant calls.

**Table 3:** Mendelian inconsistency rates per triplet (father, mother and offspring) for the genotypes by joint modeling of family data (top panel) and by the standard approach where the relatedness was ignored, i.e. individuals were treated as unrelated (bottom panel) for sequencing coverage of 5× to 30× and bases with Phred-scaled quality Q20 (1% error rate) and 30 (0.1% error rate) without mapping error.

		Mendelian inconsistency rate per triplet				
Calling method	Base quality	5×	10×	15×	20×	30×
Family-aware calls	Q20	0.00011	1.9*10^−6^	4.3*10^−8^	0	0
	Q30	5.7*10^−5^	4.6*10^−7^	0	0	0
Standard calls	Q20	0.025	0.0049	0.0011	0.00023	0.00014
	Q30	0.022	0.0029	0.00045	8.0*10^−5^	2.7*10^−6^

### Allocation of sequencing differentially to family members

To explore strategies for allocating sequence coverage among different family members, we distributed a total of 150× simulated coverage across individuals in the 3-generation pedigree. We investigated three scenarios: in the first, all individuals were sequenced to 15×; in the second, founders were sequenced deeply to 30× each and non-founders to 5× each; in the third, founders were sequenced to a depth of 6× each and the rest of the pedigree to 21×. For these three scenarios, the numbers of variants discovered differed substantially, with equal coverage of 15× for all pedigree members uncovering 0.45% and 3.3% more variants than the second and third scenario respectively. The equal coverage scenario also produced the most accurate genotypes. For example, the heterozygote mismatch rate across all individuals was 0.24% for equal coverage and 3.83% and 1.67% for the second and third scenarios; the mismatch rates at sites homozygous for the alternative allele was 0.076%, 0.85% and 0.51% for the three scenarios respectively. When we focused on genotype accuracy for rare variants with MAF<0.01, the same pattern holds. For example the heterozygous mismatch rates are 0.2%, 3.9% and 1.4% for the three scenarios respectively. Genotype accuracy of each individual is highly correlated with the depth and the mismatch rates of individual family members are displayed in Figure 1C, 1D. In these simulations, equal allocation of sequencing to founders and non-founders thus seems optimal for variant calling.

### Detection of *de novo* mutation

**Figure 3:**
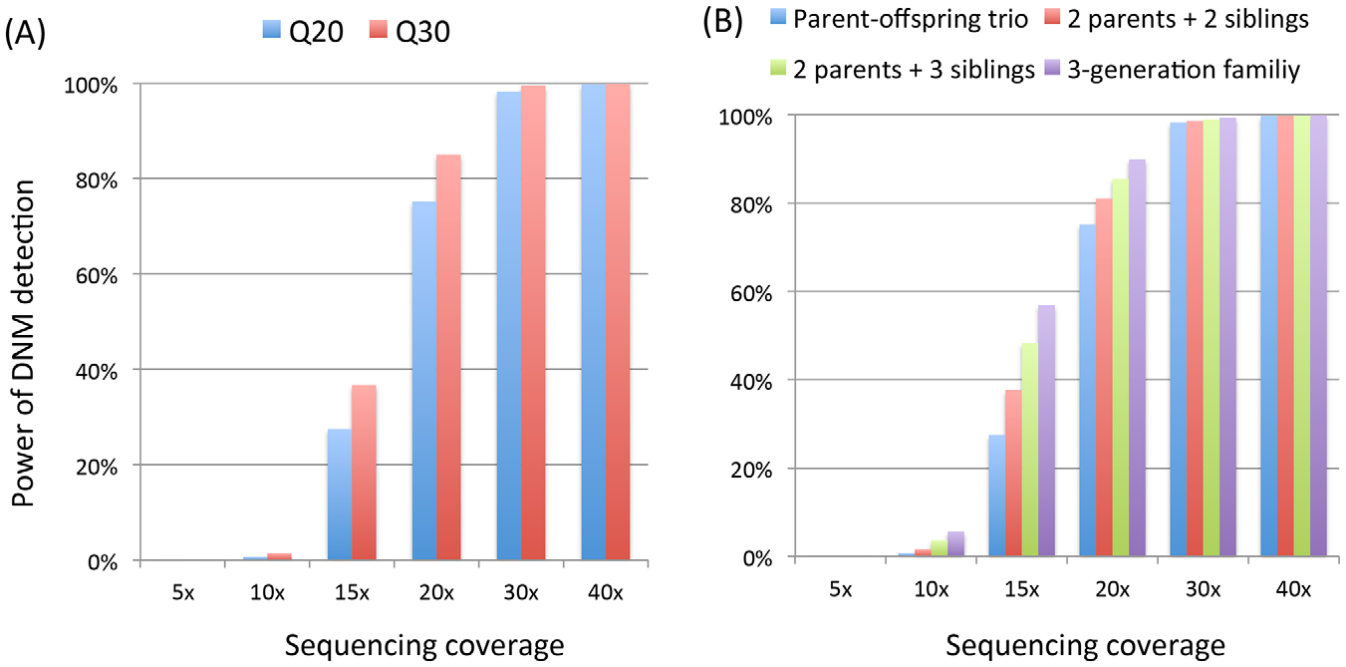
Power of detecting *de novo* mutations (DNM) in different pedigree structures for coverage from 5× to 40×. Panel A) shows the power for trios with base quality Q20 and Q30 and panel B) shows the power comparisons of trios, nuclear families with 2 and 3 siblings, and 3-generation extended pedigrees (shown in Figure 1) for base quality Q20 without mapping error.

We investigated the power to identify *de novo* mutations in trios, nuclear families where two parents and multiple siblings are sequenced, and the 3-generation pedigree. For the 3-generation pedigree we evaluated the performance on individual II-2. In this study, a *de novo* mutation was claimed when 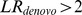 and the inferred genotypes of a triplet violate Mendelian consistency with simulated true genotypes. We found power to be extremely low for 5× and 10× coverage, in all pedigree structures considered. We found power to increase dramatically for coverage of 15× to 20× and higher (Figure 3). At about 30×, the power is over 98% and at 40× the power is close to 100% (Figure 3). As expected, for base quality of Q30, the detection rate of *de novo* mutations is higher than when bases have Q20 quality, except when depth is very low (low power) or very high (saturated signal) (Figure 3).

Since both *de novo* mutation and variant calling are integrated in the same framework, next we investigated whether examining additional related individuals could increase the power of *de novo* mutation detection. For base quality Q20, we observed that in comparisons with trios analyzed separately, sequencing additional siblings increases power for detection of mutation events, especially for intermediate coverage levels of 15×–20× (Figure 3B). For SNPs, detecting a *de novo* mutation event typically requires deeply sequencing the parents. In the pedigree in Figure 1, the detection power is greater than for nuclear families (Figure 3), because more information is available to the genotype caller, increasing the genotype accuracy in the triplet of I-1, I-2 and II-2.

To evaluate the false positive rate for *de novo* mutation detection, we applied the same calling procedure on data simulated without *de novo* mutations. It is shown that false positives are well controlled for all pedigrees and coverage levels investigated (Table 4). For example, at 30× which is the coverage where >98% *de novo* mutations can be detected, the false positives rate remains extremely low (Table 4).

**Table 4:** Number of false positive *de novo* mutations per billion bases detected by PolyMutt of jointly modeling for sequencing at coverage 5×–40× with Phred-scaled base quality Q20 (1% error rate) without mapping error in different pedigrees structures.

Coverage	Parents-offspring trio	2 Parents+2 siblings	2 Parents+3 siblings	3-Gen pedigree
5×	0	0	0	0
10×	0	0	2	0
15×	0	0	4	4
20×	0	4	1	1
30×	0	1	0	1
40×	0	0	0	0

### Effect of mapping error

Results presented so far are based on idealized simulations that ignore (for example) errors in read mapping. To evaluate the usefulness of our methods in a more challenging set of simulations, we conducted a second set of simulations where genotypes from founders were simulated using one of the 1000 Genomes samples as templates and where we simulated raw reads (rather than aligned bases). We followed the best-practice procedures (see *Simulations*) for variant calling and for the re-alignment step, we provided GATK with a list of simulated indels as candidates – which is an idealized situation but approximates what may soon be possible as variation catalogs become more complete and include >99% of the variants typically segregating in any single individual.

We compared the mismatch rate at heterozygous sites and the Mendelian inconsistency rate for genotype calls generated by our methods (implemented in PolyMutt) and a gold-standard variant caller that ignores family structure (implemented in the GATK version 1.3-5). To explore the impact of mapping error in our variant calls, we also analyzed variable sites with >10 bp apart from a nearby SNP or indel; at sites with nearby variants we expect a larger fraction of reads to map incorrectly (Table 5). The advantages of using family data were also clear in this analysis. For example, using GATK, at 15× coverage the error rate in called genotypes at heterozygote sites was 1.22% overall; when we focused on sites >10-bp away from the nearest variant, this error rate decreased slightly to 0.9%. Analyses using PolyMutt, but also ignoring family structure, produced similar error rates (Table 5). In contrast, modeling family structure reduced error rates to 0.71% and 0.51% for all variants and for variants >10 bp apart from the nearest variant, respectively; a reduction in error rate of >40% in each case. (Table 5). These per genotype error rates are also manifest in large numbers of Mendelian inconsistencies (and potential false-positive candidates for *de novo* mutation events). Using GATK, about 2.26% of sites resulted in Mendelian inconsistencies in the 3-generation pedigree at 15× coverage and 0.97% of sites at 30× coverage (Table 5). On the other hand, modeling relatedness dramatically reduced Mendelian inconsistencies to a rate <0.01% (and, thus, false positive *de novo* mutation events) (Table 5). In comparison to simulations without mapping error (summarized in Table 2), analyses that allow for mapping error show much reduced accuracy (for example, at 15× depth the average error rate for our caller was 0.71% when mapping error was simulated versus 0.24% when it wasn't), but still show a notable advantage for modeling family structure.

**Table 5:** Heterozygous mismatch rates (%) and Mendelian inconsistency rates (%) per site of call sets generated by PolyMutt (family-aware) and the standard approaches using PolyMutt (ignoring relatedness) and GATK from empirically calibrated alignments of simulated reads with base quality of Q20 in the pedigree shown in Figure 1.

		SNPs				Indels			
		Heterozygote mismatch rate (%)		Mendelian error rate per site (%)		Heterozygote mismatch rate (%)		Mendelian error rate per site (%)	
Coverage	Calling strategy	All variants	Variants >10 bp apart	All variants	Variants >10 bp apart	All variants	Variants >10 bp apart	All variants	Variants >10 bp apart
15×	GATK	1.22	0.90	2.26	1.82	5.96	1.93	7.66	3.32
	Polymutt(ignoring relatedness)	1.24	0.91	2.00	1.55	6.05	1.98	7.61	3.19
	Polymutt(family-aware)	0.71	0.51	<.01	<.01	4.07	1.24	<.01	<.01
30×	GATK	0.67	0.35	0.97	0.53	5.45	1.00	6.22	1.48
	Polymutt(ignoring relatedness)	0.69	0.36	0.91	0.47	5.54	1.03	6.26	1.46
	Polymutt(family-aware)	0.45	0.24	<.01	<.01	3.77	0.71	<.01	<.01

### Analysis of short insertion deletion polymorphisms

Analysis presented so far focused on single nucleotide polymorphisms, the most abundant class of variants in the genome. Many variant callers can now call other types of variants and some, such as GATK, can calculate genotype likelihoods for each examined site (summarizing the likelihood of observed reads for different, true underlying genotypes). To explore the utility of our framework for the analysis of other types of variants, we next performed indel calling using GATK and then used PolyMutt to refine the calls taking into account family information and GATK generated genotype likelihoods. In this analysis, we evaluated genotype accuracy only at indel sites where the called reference and alternate alleles matched the underlying simulation, skipping over sites where the alternate alleles were called incorrectly. It is clear that the genotyping error rates at indel sites are much higher than for SNPs (Table 5). For example, the GATK heterozygote error rate at 15× coverage was 5.96% (compared to 1.22% for SNPs). Indel calling improved substantially when we focused on sites separated by >10-bp from the nearest variant, but even then the heterozygote error rate was still 1.93% (Table 5). Again, modeling relatedness using PolyMutt reduced error rates by 30–40% (Table 5). This increased genotyping accuracy was also manifest in reduced Mendelian inconsistency rates. For example, Mendelian error rates are <0.01% at variant sites with >10 bp apart for PolyMutt calls modeling relatedness in comparison to 3.32% and 1.48% for 15× and 30× GATK calls that ignore family structure (Table 5). We also called genotypes using PolyMutt assuming family members are unrelated and the results are similar to GATK for both genotype accuracy and Mendelian inconsistency (Table 5).

### Detection of *de novo* mutations in the presence of mapping error

**Figure 4:**
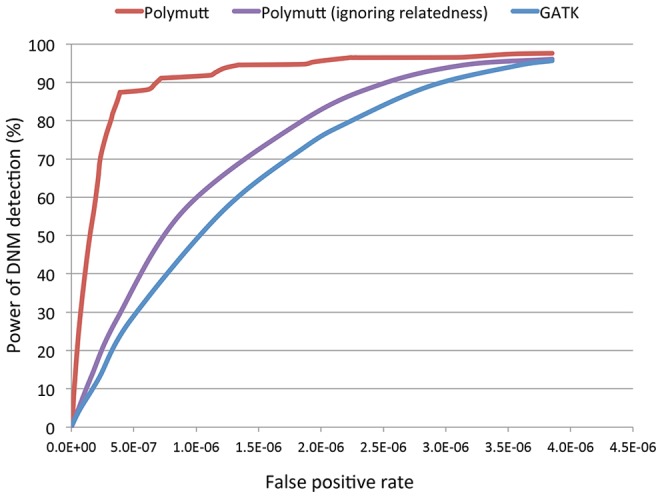
The receiver operating characteristic (ROC) curves of PolyMutt and the standard methods for *de novo* mutation (DNM) detection from empirically calibrated alignments of simulated reads with sequencing coverage of 30× with base quality of Q20. PolyMutt (ignoring relatedness) and GATK calls were obtained by jointly calling a trio assuming individuals in a trio are unrelated using Polymutt and GATK respectively.

After demonstrating that modeling relatedness could improve detection and genotyping of SNPs and indels, even in the presence of mapping error, we proceeded to evaluate the impact of mapping error on detection of *de novo* mutation events. We simulated *de novo* mutations at a rate of 1.4×10^−8^ per bp per generation in trios sequenced at 30×. As in the previous sections, we based founder genotypes on those observed in the 1000 Genomes Project and, after read mapping, re-alignment and recalibration called *de novo* mutations either by analyzing trio assuming individuals are independent using GATK's standard caller (followed by a series of filters shown to help reduce the number of spurious Mendelian inheritance errors [18]) or by using the methods described here and implemented in PolyMutt. In addition, we also used PolyMutt to detect *de novo* mutations assuming individuals are unrelated, to compare with the GTAK calls. We called a candidate *de novo* event only in places where the parents were called as homozygotes for the reference allele, and the offspring was called as heterozygote [18]–[20] – a strategy that removes most alignment artifacts. We varied the likelihood ratio values (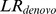) for PolyMutt calls and the minimum depth in a trio for standard calls to tune false positive and false negative rates. Using the above criteria, we calculated the power and false positive rates for the Polymutt call set, and also the two call sets generated by GATK and PolyMutt ignoring relatedness. The ROC curves for the three call sets are displayed in Figure 4. We can see that PolyMutt clearly outperforms standard approaches and a power of 90% is quickly reached at a false positive rate of 6.7*10^−7^ (Figure 4). On the other hand, analyses that did not model family structure resulted in much higher false positive rates. For example, achieving 80% power in this simulated dataset required allowing for a false-positive rate of 3.3*10^−7^ when family structure was modeled, but 2.2*10^−6^ and 1.9*10^−6^ for calls from GATK and PolyMutt ignoring relatedness, corresponding to an increase in false positives of ∼6–7 fold (Figure 4).

### Application to real data for variant calling

**Figure 5:**
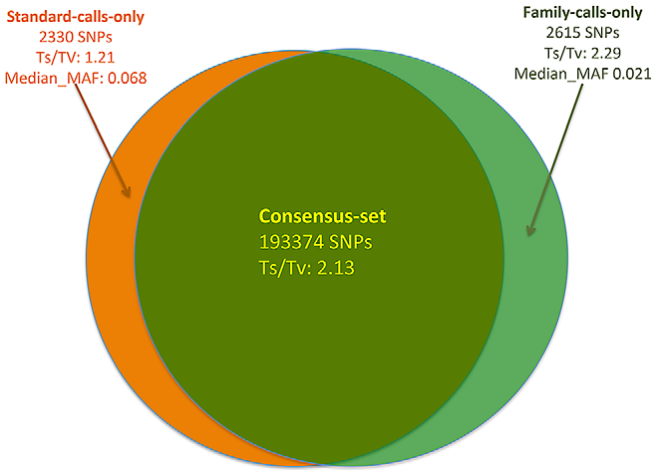
Comparisons of two variant callsets from SardiNIA low-pass sequencing data where variant calling was carried out by explicitly modeling family relatedness (family calls) and by the standard approach of ignoring relatedness (standard calls).

We are currently sequencing related individuals in Sardinia to study the genetics of immune and aging related traits [40], [41]. We applied our framework on an early data freeze totaling 66 samples and including 13 trios, four parent-offspring pairs, four families with two siblings with one parent, and 7 unrelated individuals. Samples were sequenced at average depth of 2–3×. Since we do not know whether called variants are true or false, except for those previously reported by the 1000 Genomes Project or in dbSNP, we used the transition/transversion (Ts/Tv) ratio to compare the quality of rediscovered dbSNPs and newly discovered SNPs. Prior research indicates that on the genome level the Ts/Tv ratio is around 2.2 and that significantly lower values can indicate an excess of false SNPs. Variant calling was carried-out in two ways: (1) jointly modeling family data and (2) using the standard approach in which relatedness was ignored. For variants detected exclusively by either approach, the Ts/Tv ratio of 2.29 in family calls is on the same level as the ratio estimated from known variants, indicating the high accuracy of variants unique to the family-aware call set. On the other hand, variants exclusively called by the standard approach of ignoring relatedness have a much reduced ratio of 1.21, closer to the expected ratio of 0.5 assuming that genome changes are random. This further indicates that ignoring family relationships results in sub-optimal use of available information. In addition, family calls are especially more accurate for rare variants. For example, the median MAF of variants only detected by family calls is roughly 3 times lower than the corresponding MAF of variants exclusive to the standard calls (Figure 5). Since the data we analyzed do not consist of complete families and some subjects are unrelated, we expect a further boost of variant calling accuracy using families when the full family data set is available.

## Discussion

Family designs were largely ignored in the GWAS era due to their decreased efficiency compared to case/control studies for common variant association studies. In the sequencing era, family designs may provide an attractive approach for studies of low frequency variants. Another attraction of family samples comes from the possibility of studying *de novo* mutation, which trio sequencing has shown may be involved in the etiology of neurological disorders [13], [16]–[20]. Current methods for calling variants and individual genotypes, typically do not model relatedness, resulting in loss of efficiency. Our simulations show that, when next generation sequencing is applied to multiple related individuals, better variant site lists and genotypes can be generated. This is particularly true for rare variants, for which the genotype accuracy is greatly increased if family relatedness is modeled in our methods (Figure 2). The advantages extend to many aspects of variant calling, including both sensitivity and specificity. For sequencing studies that aim to identify *de novo* mutations, our results illustrate the advantages of modeling all family members simultaneously. For standard methods, false positive *de novo* mutations can be due to false negative variant calls in parents. Our methods model polymorphism and *de novo* mutation simultaneously in the same framework and increase both the sensitivity and specificity when family information is available. Similar calling algorithms for *de novo* mutations have been proposed for trios [42], [43] and our methods now extend the analysis of next generation sequence data to arbitrary pedigrees.

Our methods are based on the Elston-Stewart algorithm for pedigree likelihood calculation, which can handle very large pedigrees without inbreeding. Marriage loops and/or inbreeding pedigrees are well known to challenge most implementations of the algorithm. Our implementation uses a well-known workaround for handling inbred pedigrees, based on loop-breaking approach [44]–[46]. To investigate the performance of this strategy, we simulated 15× sequence data for 3-generation first-cousin marriage pedigrees with 10 individuals. We performed variant calling using PolyMutt by the loop-breaking approach and also the standard approach assuming that individuals are unrelated. The overall genotype mismatch rate for the standard approach is 0.11%, compared to 0.049% for the family joint calls. When we focused on heterozygotes, the mismatch rates are 0.51% and 0.24% respectively. These statistics are very similar to the results shown for the 3-generation pedigree in Table 2 and demonstrated that although there is loss of information by loop-breaking, modeling relatedness remains highly useful.

Current protocols for deep genome and exome re-sequencing typically aim for coverage >30× and we thus expect excellent accuracy of genotype calls and power to identify *de novo* events in many existing datasets. Still, even in these cases, our methods provide substantial improvements in the accuracy of genotype calls – particularly in our more realistic simulations that allow for mapping error and examine hard to call variants (such as indels or variants that are clustered with other nearby polymorphisms). Furthermore, in some regions, depth may be lower (e.g. due to unusual GC content or the vagaries of exome capture) and in these regions analysis will continue to benefit from modeling of familial relationships even when the average depth for the rest of the genome leaves most genotypes beyond doubt. It may also occur that individuals in a family are sequenced at different coverage, using different platforms, with varying read lengths and sequencing error rates, and other systematic differences. For example offspring may be sequenced later with technologies that produce higher coverage and lower error rates. By jointly modeling related individuals in pedigrees, our methods can improve the accuracy of genotype calls for individuals sequenced earlier.

We first conducted an initial set of simulations that ignored mapping error. These data were efficient to simulate and analyze, allowing us to explore a variety of family configurations, sequencing error rates, and to vary depth of coverage. In a second set of simulations, we used founder haplotypes from the 1000 Genome Project and simulated short reads that were then mapped and processed as in standard analysis for several ongoing projects. Error rates were noticeably higher than in ideal situations assuming no mapping error. This increase is expected, since sequence alignment error is one of the major sources for variant calling problems [1]. For example, at 30× depth heterozygous genotyping error increased from 0.006% (with no mapping error) to ∼0.67% (with mapping error simulated) when family information was ignored in the 3-generation pedigree. Modeling family information reduced these error rates to 0.0025% (with no mapping error) and ∼0.45% (with mapping errors), respectively. Family-aware calling using the methods described here greatly increased genotyping accuracy and reduced Mendelian inconsistency rates. Mapping and alignment challenges have a far more severe effect on more complex variants, such as short insertion deletion polymorphisms. Typically, reads containing these variants are harder to align and interpret correctly. Still, just as for SNPs, our methods appear to reduce the genotyping error rate significantly and of particular note to essentially eliminate Mendelian inconsistencies.

For a fixed sequencing effort, our simulations suggest that it will be optimal to distribute sequencing coverage equally among family members. However it is not trivial in general to determine the optimal allocation of sequencing to families for association studies. Although larger sample sizes have better power to identify genetic factors, when a fixed sequencing budget is distributed among more samples, genotyping accuracy can be compromised. The power of association studies is dependent upon the interplay between the sample size, the pedigree structure and the genotype accuracy. In unrelated individuals the variant discovery rate is optimal when sequencing many individuals, each with somewhat lower coverage [1], [47] and we expect this will also hold true for family studies. One question that remains to be addressed is how to determine the optimal allocation of sequencing to families in order to obtain the best power for association studies. Besides the sequencing quality, the factors to consider include number of families, family size, family structures and selection for specific disease status or trait values. This important issue is beyond the scope of the current study and will be investigated elsewhere.

*De novo* mutations have been implicated to play an important role in some complex diseases, such as autism. Sequencing allows for efficient detection of *de novo* point mutations and has begun to reveal potential causal genes for sporadic autism [18]–[20]. Most of studies focused on the trio design due to its simplicity. Sequencing unaffected siblings could provide valuable information about background mutation within family, and we expect these studies to soon extend into quartet families (where background mutation can be examined) or even extended pedigrees (where the parental origin of mutations can be examined). For the simple trio design, we demonstrated the advantages of our methods over standard approaches. For more complex pedigrees, our methods will continue to improve the sensitivity and specificity due to the use of sequencing data from the entire family, as shown in Figure 3, and will further outperform standard approaches of analyzing individual trios.

In unrelated individuals, modeling of linkage disequilibrium is a very effective strategy for improving the accuracy of genotype calling [47]. In principle, it would be ideal to combine the haplotype-based imputation with the constraints of Mendelian inheritance in pedigrees for variant calling. However, this process remains very challenging computationally because integrating over haplotype distributions for multiple founders increases the complexity of the problem exponentially. We are actively investigating heuristic solutions to the problem, and hope to report on them in the future.

In this study, we developed a likelihood framework for variant and genotype calling and *de novo* mutation detection in families from sequencing data, and showed its superior performance compared to the standard approach in which family members are treated as unrelated. We have implemented the framework in PolyMutt, a software package for Polymorphism and Mutation detection, which can be downloaded from authors' website (http://sph.umich.edu/csg/bingshan). There is a growing interest in using family sequencing data for genetic studies and we hope that our methods will be helpful for identifying genetic factors associated with human diseases by providing accurate variant and genotype calls and *de novo* mutations in family sequencing studies.
